# An Unusual Recurrence of Signet Ring Cell Gastric Adenocarcinoma Treated by Right Hemicolectomy, Pancreaticoduodenectomy, and IVC Resection: Controversies and Dilemmas of Following Standard Treatment Pathways

**DOI:** 10.7759/cureus.424

**Published:** 2015-12-21

**Authors:** Mikael Sodergren, Kirsty Brammer, Patrick L Zentler-Munro, David Cunningham, Satvinder Mudan

**Affiliations:** 1 Department of Surgery and Cancer, Imperial College London; 2 Department of Academic Surgery, The Royal Marsden NHS Foundation Trust; 3 Retired Consultant Gastroenterologist, Faringdon, Oxfordshire; 4 Department of Gastrointestinal Oncology, The Royal Marsden NHS Foundation Trust

**Keywords:** signet ring cell cancer, adrenocortical carcinoma, metastases, solitary, recurrence

## Abstract

We present the case of a 67-year-old male patient with a past history of previously resected T3 right adrenocortical carcinoma and T3N1 signet ring cell adenocarcinoma of the stomach who presented with recurrence of gastric cancer in the form of a large solitary mass in the right abdomen. He was treated with ECX (epirubicin, cisplatin and capecitabine) chemotherapy and multivisceral resection. This recurrence pattern is the first such description in the literature, and we discuss the controversies and arguments in favour of offering surgical resection.

## Introduction

Linitis plastica, also known as signet ring adenocarcinoma of the stomach, although rare but rising in incidence, is associated with a poor prognosis and high recurrence rates following surgical resection. It accounts for about 10% of all gastric cancers and is characterised by rigidity and fibrotic thickening of the stomach wall. Standard treatment pathways are those of gastric cancer, and surgical resection remains the only recognised therapeutic option offering a potential cure. Recurrence is often in the form of disseminated peritoneal disease.

## Case presentation

We report the case of a 67-year-old retired consultant gastroenterologist with a past medical history of primary hypothyroidism and past surgical history of a right adrenalectomy and nephrectomy with inferior vena cava (IVC) resection in 2008 for a pT3 high-grade adrenocortical carcinoma. Complete resection of the tumour was achieved microscopically with free-floating tumour extension into the intrahepatic inferior vena cava. The tumour was ruptured intraoperatively, and he was therefore advised to start long-term adjuvant mitotane. This caused chronic adrenal insufficiency, treated with replacement therapy, and significant hypercholesterolaemia. This is believed to have lead to a symptomatic superior mesenteric artery stenosis presenting in 2010, which was successfully treated with a superior mesenteric artery stent.

**Figure 1:**
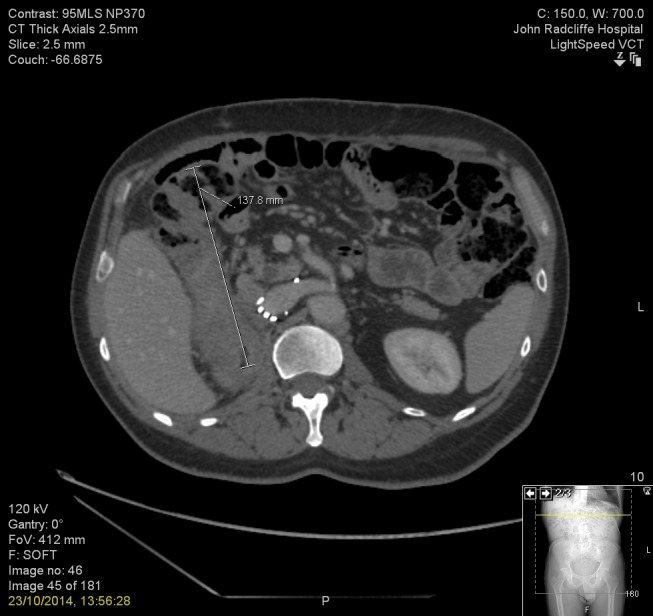
CT images showing mass centered on ascending colon and hepatic flexure.

In January 2012, he presented with melaena and was found to have a distal antral gastric carcinoma, infiltrating submucosally, biopsies of which were consistent with linitis plastica. He went on to have a subtotal gastrectomy, cholecystectomy, and D2 lymphadenectomy with neo-adjuvant and adjuvant ECF chemotherapy (3 + 2 cycles). The 5-FU was replaced by raltitrexed due to minor asymptomatic coronary disease and the sixth cycle was omitted as he moved to a different region of the country. Histopathology showed a poorly differentiated diffuse type adenocarcinoma of the antrum, staged as pT3 N1 (1/27) V0 R0, Mandard TRG 3. The mitotane was stopped at the start of chemotherapy as no recurrence of the adrenocortical carcinoma was evident on follow-up imaging. He remained well until August 2014 when he developed early satiety and underwent gastroscopy, which showed no macroscopic abnormality, and random biopsies from the staple line showed no malignancy. The patient presented again in October 2014 when he developed further abdominal pain. Computed tomography cross-axial imaging showed a 14 cm mass around the ascending colon and hepatic flexure with a suspicion of retroperitoneal and psoas involvement, as illustrated in Figure 1. Prior to the symptom-induced imaging diagnosis of recurrence in October 2014, the previous adrenal follow-up imaging in February of that year was reported as normal. Review, however, suggested that there may have been an area of thickening in the wall of the colon at the hepatic flexure.

A tunnelled transcolonic biopsy of this mass revealed the recurrence of the gastric adenocarcinoma. Further imaging in the form of a PET scan in December 2014 showed moderately FDG-avid (maximum SUV 9.2) wall thickening of the large bowel at the hepatic flexure extending over a length of 10 cm, abutting the right psoas muscle; the uptake in the muscle was thought to be physiological.

The case was discussed at the referring hospital's multi-disciplinary team meeting (MDT), and he was offered six courses of palliative EOX chemotherapy. At the patient’s request, he was also referred to our institution for a second opinion. He was subsequently seen in the oncology and surgical clinics and discussed a number of times at our regional MDT. On review of further imaging from March 2015, it was felt that the edge of the mass was not infiltrating the psoas fascia and that the retroperitoneal fascia was intact, taking account of the postoperative changes from his previous adrenal surgery. It was also noted that there was encasement around the pancreas and focal dilatation of the distal common bile duct due to a mesenteric invasion, although there was no significant derangement in liver function tests. Following extensive discussion, the final suggestion was for the patient to be offered surgical resection after completing six cycles of EOX (with no significant radiological response). Informed patient consent was obtained. No identifying patient information is disclosed in this paper. 

On July 22, 2015, the patient underwent a right hemicolectomy with en bloc pancreaticoduodenectomy, IVC resection, and retroperitoneal lymphadenectomy. The histology reported that sections showed the presence of fibrosis in the wall of the colon and in areas of adhesion between the large and small bowel. Within the fibrous area, there was an uneven infiltrate of poorly differentiated adenocarcinoma with diffuse/signet ring cell morphology. The carcinoma was present in the submucosa and muscularis propria of the colon as well as the surrounding fibrous tissue of the adhesion, but there was no definite lymphatic or venous invasion. No tumour was seen in the four lymph nodes identified and the area of infiltration appeared completely excised. Immunohistochemistry highlighted that the tumour also extended into the colonic mucosa, and was negative for HER2. The patient made an uneventful recovery, interrupted only by admission with an intra-abdominal collection, successfully treated with percutaneous drainage. Follow-up CT in November 2015 showed no evidence of disease recurrence.

## Discussion

Recurrence from gastric linitis plastica tumours is often in the form of the disseminated peritoneal disease and standard treatment pathways are those of gastric adenocarcinoma. However, there is increasing evidence that linitis plastica tumours of the stomach should be considered a different disease entity from non-linitis plastica adenocarcinoma [1]. Furthermore, the optimal chemotherapeutic regimes are still controversial, as most studies do not differentiate between linitis plastica and non-linitis plastica patient groups [2-3]. It is also important to identify hereditary diffuse gastric cancer [4]. This patient has first and second male cousins both diagnosed with breast cancer and his father had bowel cancer at the age of 72, but clinical genetics evaluation found no significant abnormalities.

It is clear that the timing and location of the lesion would have been more likely to represent a recurrence of the adrenocortical carcinoma, and this highlights the importance of histological confirmation. There are, moreover, several atypical features of this pattern of linitis plastica recurrence that should be highlighted. As mentioned, review of earlier imaging suggested that there might have been an area of thickening in the wall of the colon at the hepatic flexure prior to the detection of the mass. This suggests that the recurrent lesion may have started in the colon rather than the retroperitoneum (as supported by the postoperative histology). Secondly, the presence of a large mass after only eight months without any obvious other sites of disease is not a characteristic pattern of recurrence for linitis plastica tumours. For haematogenous metastases of linitis plastica, there is no proven role for surgery, and this was the justification for the treatment recommendation from the referring hospital, which in itself appears evidence-based. In this case, however, there was no evident disease in more proximal sites for the haematogenous spread, such as the liver or lung, raising the question of whether this represented a local recurrence. The fact that the recurrence was solitary and had remained solitary for a reasonable period of time, in addition to the atypical location for this histological tumour type, were significant factors contributing to the decision to offer surgical resection. A final interesting consideration in this case, although less likely, is whether this tumour could represent a third primary, as primary linitis plastica of the colon has been reported [5]. The patient, in his own right a specialist in this pathology having completed a career in clinical gastroenterology, recognised certain anomalies and requested a second opinion. Nevertheless, the decision to proceed to surgical resection was not straightforward and our MDT recognised that there was a chance that it might not be technically possible to achieve an R0 resection or that there would be peritoneal disease detected intra-operatively. It was felt that including a multivisceral resection in the form of pancreaticoduodenectomy would be the best chance of achieving a negative margin. At surgery, an en bloc inferior vena cava resection was performed, as there were some thickening and nodular attachments from the main mass.

## Conclusions

This specific recurrence pattern represents, to our knowledge, the first such case described in the literature. The fact that surgical clearance required a major multivisceral resection with significant potential morbidity, coupled with the uncertain efficacy of palliative chemotherapy, highlights the dilemma faced by clinicians in advising the most appropriate treatment strategy. All such cases should be discussed in a specialist MDT and any surgical intervention should be reserved for patients who are fully informed of the available evidence base, morbidity profile associated with the surgery, and rationale for such treatment.
